# Pandemic Influenza and Healthcare Demand in the Netherlands: Scenario Analysis

**DOI:** 10.3201/eid0905.020321

**Published:** 2003-05

**Authors:** Marianne L.L. van Genugten, Marie-Louise A. Heijnen, Johannes C. Jager

**Affiliations:** * National Institute for Public Health and the Environment, Bilthoven, the Netherlands

**Keywords:** scenarios, pandemic influenza, preparedness planning, outbreak control, research

## Abstract

In accordance with World Health Organization guidelines, the Dutch Ministry of Health, Welfare and Sports designed a national plan to minimize effects of pandemic influenza. Within the scope of the Dutch pandemic preparedness plan, we were asked to estimate the magnitude of the problem in terms of the number of hospitalizations and deaths during an influenza pandemic. Using scenario analysis, we also examined the potential effects of intervention options. We describe and compare the scenarios developed to understand the potential impact of a pandemic (i.e., illness, hospitalizations, deaths), various interventions, and critical model parameters. Scenario analysis is a helpful tool for making policy decisions about the design and planning of outbreak control management on a national, regional, or local level.

In 1997, avian influenzavirus was shown to infect humans directly when an influenza virus A/H5N1 infected 18 people in Hong Kong; of those, six died (*1*,*2*). After this event, experts predicted that another influenza pandemic is highly likely, if not inevitable (*3*,*4*). The impact of a pandemic depends on factors such as the virulence of the pandemic virus and the availability of a vaccine. Because development is time-consuming, the vaccine would likely not be available in the early stages of a pandemic, and a major vaccine shortage would be expected (*5*). An influenza virus pandemic would likely cause substantial social disruption because of high rates of illness, sick leave, hospitalization, and death. Therefore, pandemic planning is essential to minimize influenza-related illness, death, and social disruption (*5*,*6*).

In accordance with World Health Organization guidelines, the Dutch Ministry of Health, Welfare and Sports developed a national plan to minimize or avert effects of pandemic influenza. Within the scope of the Dutch pandemic preparedness plan, we were asked to estimate the magnitude of the problem in terms of the expected number of hospitalizations and deaths during an influenza pandemic. We also estimated the potential effects of intervention options, including the use of the relatively new antiviral drugs, neuraminidase inhibitors (*7*,*8*).

One published study (*9*) has estimated the economic effects of an influenza pandemic. Meltzer et al. examined the possible effects of influenza vaccine-based interventions in terms of outpatient visits, hospitalizations, deaths, and related costs during a pandemic in the United States. More recently, different strategies for the control of interpandemic influenza for the elderly population in three European countries (England and Wales, France, and Germany) have been evaluated (*10*). Our objective was to examine the potential impact of pandemic influenza in the Netherlands and to analyze the effects of several (other than influenza vaccine–based) possible interventions in terms of hospitalizations and deaths.

## Methods

Predicting when the next influenza pandemic will occur and how it will evolve is impossible, and the same is true for forecasting the number of persons who will become ill, be hospitalized, or die. Because of the many uncertainties, we performed a scenario analysis (*11*) that included consulting of experts and modeling. At a meeting of experts held to discuss an influenza pandemic in the Netherlands, specialists on influenza (virology, epidemiology, and surveillance) and on controlling epidemics and disasters gave their opinions about the formulated intervention scenarios, the assumptions made, and the value of critical parameters (*12*). A model was used to estimate the number of hospitalizations and deaths in the Netherlands for different scenarios. We also compared the number of expected hospitalizations and deaths for each of the different intervention scenarios to the number expected for the nonintervention scenario.

### Scenarios

Various scenarios are possible, depending on whether influenza vaccine, pneumococcal vaccine, or antiviral drugs are available (among other factors). In all scenarios, we assumed a gross attack rate of 30%; we also assumed age-specific attack, hospitalization, and death rates and healthcare utilization (e.g., antibiotic drug prescription) as in a regular epidemic. Table 1 shows the base-case assumptions in the various scenarios. Following are descriptions of the scenarios considered relevant and sufficiently realistic by the specialists who participated in the meeting of experts.

**Table 1:** Assumptions made for influenza pandemic scenario analysis, the Netherlands

Scenario	Assumptions in base case	Assumptions in sensitivity analysis
No intervention	Gross attack rate of 30%; age-specific attack, hospitalization, and death rates as in regular epidemic; and healthcare utilization as in regular epidemic.	Gross attack rate of 10% and 50%; age-specific attack rates (see Table 4); and complication rates for a) persons <64 y of age x 2 and b) persons at low risk equal to persons at high risk.
Influenza vaccination of risk groups (including persons >65 y of age) and healthcare workers	Gross attack rate of 30%; age-specific attack, hospitalization, and death rates as in regular epidemic; and vaccine efficacy 80% (<64 y of age) (*13*,*14*) and 56% (>65 y) (15) to prevent hospitalizations and deaths	Gross attack rate of 10% and 50%; age-specific attack rates (see Table 4); complication rates for a) age group <64 y times 2 and b) persons at low risk equal to persons at high risk; influenza vaccine efficacy a) 80% for all ages and b) 40% for age group <64^a^ and 30% for age group >65^b^.
Pneumococcal vaccination of influenza of risk groups (including persons aged >65 y)	Gross attack rate of 30%; age-specific attack, hospitalization, and death rates as in regular epidemic; 50% pneumococcal-related hospitalizations; and vaccine efficacy 64% against invasive infections (*16*,*17*).	Gross attack rate of 10% and 50%; age-specific attack rates (see Table 4); complication rates for a) persons <64 y of age x 2 and b) persons at low risk equal to persons at high risk; 25% and 75% pneumococcal-related hospitalizations; and vaccine efficacy 25% and 75%.
Therapeutic use of neuraminidase inhibitors for all patients with influenzalike illness	Gross attack rate of 30%; age-specific attack, hospitalization, and death rates as in regular epidemic; and 50% reduction of hospitalizations and deaths.	Gross attack rate of 10% and 50%; age-specific attack rates (see Table 4); complication rates for a) persons <64 y of age times 2 and b) persons at low risk equal to persons at high risk.; and 25% to 75% reduction of hospitalizations and deaths.

^a^Minimum variant based (*9*). **^b^Maximum variant assumes 80% efficacy for all ages**

### Nonintervention Scenario

The nonintervention scenario is a “worst case” situation in which no intervention is possible. The scenario includes a pandemic influenza for which no vaccine is available and only regular care and regularly prescribed antibiotic drugs are provided. In the base case, we assume a gross attack rate of 30%; an age-specific attack; and hospitalization, death rates, and healthcare utilization as in a regular epidemic.

### Influenza Vaccination Scenario

In this scenario, when an influenza vaccine becomes available, two possible strategies are considered: 1) vaccination of risk groups including persons >65 years of age (n = 2.78×10^6^) and healthcare workers (n = 0.80×10^6^) and 2) vaccination of the total population (n = 15.6×10^6^). In the base case, influenza vaccination is assumed to be 56% effective in preventing hospitalizations and deaths in persons >65 years of age (*15*), and 80% effective in those <64 years of age (Table 1) (*13*,*14*).

### Pneumococcal Vaccination Scenario

In the absence of a vaccine available at the beginning of a pandemic, the Dutch Health Council recommends providing influenza risk groups (including those >65 years of age; n = 2.78×10^6^) with pneumococcal vaccination (*18*), which is a 23-valent vaccine assumed to prevent invasive infections caused by *Streptococcus pneumoniae*, one of the possible complications of influenza. For the base case, we assumed that 50% of hospitalizations and deaths from influenza-related pneumonia are caused by invasive pneumococcal infection and that pneumococcal vaccination prevents 80% of invasive infections caused by vaccine serotypes (Table 1) (*16*,*17*). In the Netherlands, 80% of serotypes involved in invasive pneumococcal infections are covered by the 23-valent vaccine, which results in a vaccine effectiveness of 64% against invasive pneumococcal infections.

### Therapeutic Use of Neuraminidase Inhibitors Scenario

This scenario includes the use of neuraminidase inhibitors. When taken within 48 hours after onset of symptoms and continued for 5 days, neuraminidase inhibitors (zanamivir and oseltamivir) (*19*) reduce the duration and seriousness of influenza by 1 to 2 days for adults (*20*–*24*), children (*22*,*25*,*26*), and persons at high risk (*22*,*27*–*29*). However, the effectiveness of neuraminidase inhibitors for preventing hospitalizations and deaths (our outcome parameters) is unknown. Therefore, we assumed that 25% to 75% of the hospitalizations and deaths attributed to influenza would be avoided by therapeutic use of neuraminidase inhibitors (*12*) in this scenario (each person with an influenzalike illness begins the medication within 48 hours after the first symptoms). An advantage of therapeutic use of neuraminidase inhibitors is that antibodies are formed (*26*) because infection is not prevented; thus protection against an infection resulting from the same virus is built up, as in an untreated infection.

Although neuraminidase inhibitors have proven to be effective prophylactically (*27**,**30**–**32*)*,* the specialists were unanimous in their opinion that using neuraminidase inhibitors prophylactically on a large scale in a pandemic is not feasible because they need to be taken as long as the threat of influenza virus infection lasts. The medication would therefore need to be taken for at least several weeks to several months in a pandemic. An enormous stockpile of neuraminidase inhibitors would be required for the Dutch population; compliance, in the course of time, would likely diminish. In this scenario, using this medication for prophylactic purposes might merely postpone the pandemic, and the disease might emerge at the moment that most of the population stops the prophylaxis unless an effective and safe vaccine is available in sufficient amount at that time.

The specialists considered neuraminidase inhibitors to be more suitable than previous antiviral medicines (amantadine and rimantadine), which lead to viral resistance, have serious side effects, and are only effective against influenza A (*7*,*8*,*14*). Neuraminidase inhibitors are effective against influenza A and B and have not generated much resistance thus far (*19*,*33*,*34*); they appear to be safe and have seldom caused serious side effects (*34*–*36*).

### Model and Data

**Figure 1:**
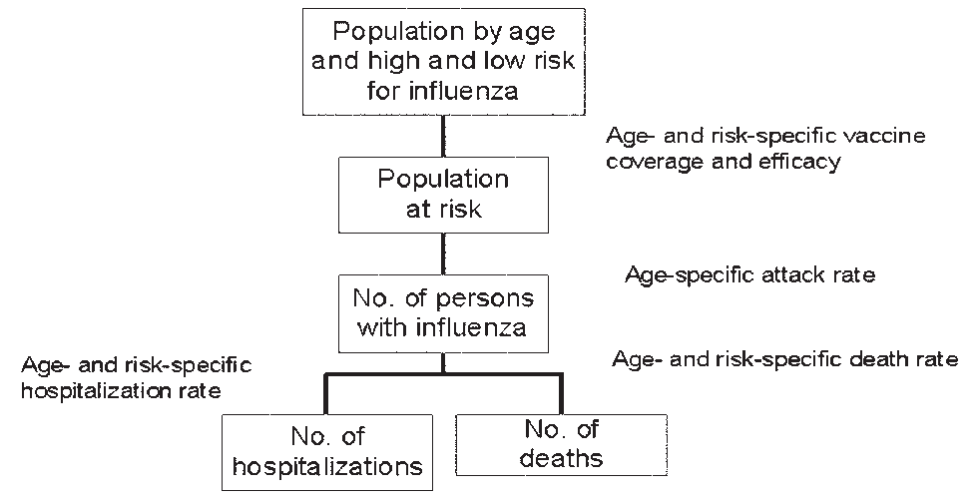
Schematic view of calculation model used for scenario analysis.

Building a mathematical model of influenza spread is difficult because of yearly differences in virus transmission and virulence, lack of understanding of the factors affecting the spread of influenza, and shortage of population-based data (*9*,*37*). We used a static model (*12*) that estimates the numbers of hospitalizations and deaths in the Netherlands by using data from earlier influenza epidemics and literature review. The model was implemented by using an Excel spreadsheet (Microsoft Corp., Redmond, CA) (Figure 1). In the model, we distinguished three age groups (<19 years, 20–64 years, and >65 years) by low or high risk (susceptibility to the complications of hospitalization and death) for influenza. The population not protected against influenza depends on vaccination coverage and vaccine and neuraminidase efficacy; all can be different in each scenario. We calculated the number of influenza cases in each age group at low or high risk for influenza by multiplying numbers not protected against influenza and attack rates. We calculated the absolute number of hospitalizations and deaths in each age group at low or high risk for influenza by multiplying the calculated number of influenza cases and the influenza-specific complication (hospitalization or death) rates. The case-specific complication rates in each age group at low or high risk for influenza are computed from general population–specific complication rates, current vaccination degree, and vaccine efficacy by assuming that during a regular epidemic 10% of the population becomes ill (*12*). The age distribution of the influenza cases in the general population is assumed to be equal to the age distribution of persons consulting their general practitioner for influenzalike illness. Table 2 shows the values of the basic input variables.

**Table 2:** Input variables used to calculate potential impact of influenza pandemic in terms of healthcare outcomes and the effect of various interventions, the Netherlands

Input variable	Age groups (y)			Sources
	<19	20–64	>65	
Population	3.8×10^6^	9.7×10^6^	2.1×10^6^	Statistics Netherlands
Population at high risk	0.09×10^6^	0.6×10^6^	0.7×10^6^	(*38*–*40*)
Age distribution of influenza cases	34.3	60.4	5.2	As in a regular epidemic in general practice (*41*)^a^
Current vaccination degree				(*42*,*43*)
Population at low risk	0.02	0.05	0.20	
Population at high risk	0.65	0.75	0.80	
Efficacy influenza vaccine	80%	80%	80%	(*13*–*15*)
Invasive pneumococcal infections				(*12*,*16*,*17*)
Related hospitalizations	50%	50%	50%	
Efficacy vaccine	64%	64%	64%	
Hospitalization rate (per 100,000) for influenza				As in a regular epidemic (*44*)^a^
Population at low risk	0.1	0.1	2	
Population at high risk	28	28	10	
Hospitalization rate (per 100,000) for influenza-related pneumonia				As in a regular epidemic (*44*)^a^
Population at low risk	0.3	0.3	38	
Population at high risk	72	72	175	
Death rate (per 100,000)				As in a regular epidemic (*45*)^a^
Low risk population	0.6	0.6	26.2	
High risk population	29.6	29.6	84.9	

^a^Assuming that during a regular epidemic 10% of the population becomes ill.

### Sensitivity Analyses

Sensitivity analyses were performed on the gross attack rate, age-specific attack, hospitalization and death rates, and on efficacy of vaccines and neuraminidase inhibitors. Table 1 describes assumptions used in sensitivity analysis.

## Results

**Figure 2:**
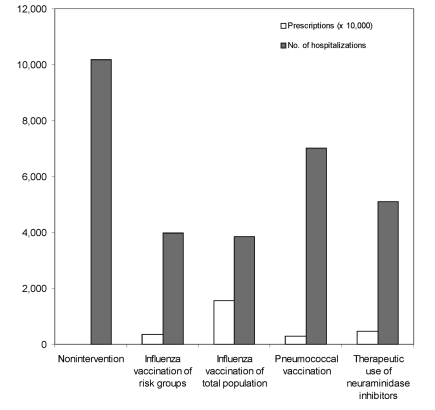
Number of hospitalizations and required prescriptions in the various scenarios.

**Figure 3:**
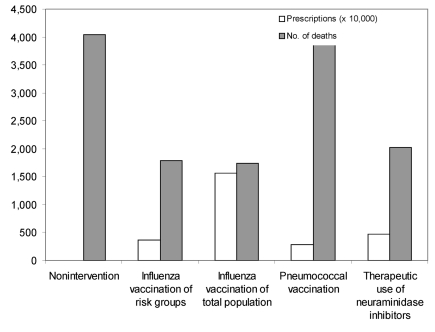
Number of deaths and required prescriptions in the various scenarios.

Results are shown in terms of number of hospitalizations and deaths (prevented) in relation to doses of vaccines or antiviral drugs needed. During a regular influenza epidemic in the Netherlands, approximately 1,900 hospitalizations and 800 deaths related to influenza occur. The nonintervention scenario of an influenza pandemic with a gross attack rate of 30% and no interventions available could lead to as many as 10,000 influenza-related hospitalizations and >4,000 deaths (Figures 2 and 3).

The influenza vaccination scenario could prevent >6,000 (>60%) of hospitalizations and >2,200 (>55%) of deaths. Vaccination of the total population requires 15.6 million doses of vaccine; vaccination only of risk groups for influenza (including persons >65 years of age and healthcare workers) requires 3.6 million vaccines. The pneumoccoccal vaccination scenario, which requires 2.8 million doses of vaccine, could prevent 2,600 (25%) of the hospitalizations and 140 (3.5%) of the deaths. The therapeutic use of neuraminidase inhibitors scenario could prevent 5,000 hospitalizations and 2,000 deaths (assuming 50% efficacy) and would require 4.7 million prescriptions of neuraminidase inhibitors.

A decrease (increase) in the gross attack rate to 10% (to 50%) shows a similar decrease (increase) in the absolute number of expected hospitalizations and deaths. Assuming different gross attack rates does not change the percentage of hospitalizations and deaths that might be avoided in the different scenarios (Table 3). By using a range of age-specific attack rates (Table 4) for the nonintervention scenario, we estimated that the number of hospitalizations ranged from 7,500 to >19,000 and the number of deaths from 2,700 to approximately 9,000 (Table 5). The variation in the number of hospitalizations and deaths in each of the scenarios is substantial. However, assuming different age-specific attack rates leads to little difference in the percentage of hospitalizations and deaths that might be avoided by a certain intervention.

**Table 3:** Hospitalizations and deaths in the scenario analysis of influenza pandemic^a^

Scenario	No. of hospitalizations			No. of deaths		
	Base case	Gross attack rate 10%	Gross attack rate 50%	Base case	Gross attack rate 10%	Gross attack rate 50%
Nonintervention	10,186	3,395	16,977	4,040	1,347	6,733
Influenza vaccination						
Total population	3,847	1,282	6,412	1,738	579	2,896
Risk groups	3,968	1,223	6,614	1,789	596	2,981
Pneumococcal vaccination	7,008	2,326	11,679	3,903	1,301	6,505
Neuraminidase inhibitors	5,093	1,698	8,489	2,020	673	3,367

^a^Assuming gross attack rates of 10% and 50%.

**Table 4:** Alternative age-specific attack rates in scenario analysis for pandemic influenza, the Netherlands^a^

Age (y)	Age groups affected as in regular epidemic	Age groups equally affected	Age groups affected in proportion of				
			1:1:2	1:2:1	2:1:1	Previous pandemics^b^	
<19	37.4	30.0	26.4	18.5	48.3	49.3	
20–64	28.6	30.0	26.4	37.0	24.1	25.6	
>65	23.1	30.0	52.9	18.5	24.1	15.0	

^a^Gross attack rate 30%. ^b^Distribution from Meltzer et al. (9) based on previous pandemics.

**Table 5:** Hospitalizations and deaths in various scenarios for alternative age-specific attack rates^a^

Scenario	No. of hospitalizations per age group						No. of deaths per age group						
	Regular epidemic	Groups equally affected	Age group proportion						Age group proportion				
			1:1:2	1:2:1	2:1:1	Previous pandemics^b^	Regular epidemic	Groups equally affected	1:1:2	1:2:1	2:1:1	Previous pandemics^b^	
Nonintervention	10,186	12,478	19,630	9,184	10,252	7,541	4.040	5,199	9,009	3,288	4,197	2,746	
Influenza vaccination													
Total population	3,847	4,844	8,068	3,285	3,939	2,716	1,738	2,245	3,929	1,401	1,809	1,169	
Risk groups	3,968	4,962	8,171	3,410	4,058	2,840	1,789	2,294	3,972	1,454	1,860	1,222	
Pneumococcal vaccination	7,008	8,574	13,460	6,323	7,053	5,200	3,903	5,015	8,697	3,178	4,054	2,654	
Neuraminidase inhibitors	5,093	6,239	9,815	4,592	5,126	3,771	2,020	2,600	4,505	1,644	2,099	1,373	

^a^Gross attack rate 30%. ^b^Distribution from Meltzer et al. (9) based on previous pandemics.

If one assumes that complication (i.e., hospitalization and death) rates for low-risk persons are equal to the complication rates for high-risk persons, the number of hospitalizations and deaths increases dramatically. In the nonintervention scenario, we estimated >64,000 hospitalizations (>10,000 in the base case) and approximately 10,000 deaths (approximately 4,000 in the base case). The number of avoided hospitalizations ranges from almost 6,000 in the pneumococcal vaccination scenario to >45,000 in the influenza vaccination (of the total population) scenario, and the number of avoided deaths ranges from 1,000 to >6,000 (Table 6). In the scenario with influenza vaccination of risk groups, this assumption leads to a decrease in the percentage of hospitalizations and deaths that might be avoided, 21% (base case 61%) and 47% (base case 56%), respectively. In the scenario with pneumococcal vaccination of risk groups, the percentage of hospitalizations and deaths that might be avoided decreases to 9% (base case 31%) and 1% (base case 3%), respectively.

**Table 6:** Hospitalizations and deaths in various scenarios for alternative complication rates^a^

Scenario	No. of hospitalizations			No. of deaths			
	Base case	Hospitalization and death rate		Base case	Hospitalization and death rate		
		Age group <64 y x 2	Low risk to high risk rate		Age group <64 y x 2	Low risk to high risk rate	
Nonintervention	10,186	12,830	64,425	4,040	4,207	10,087	
Influenza vaccination							
Total population	3,847	4,376	16,798	1,738	1,771	3,981	
Risk groups	3,968	4,617	50,935	1,789	1,873	5,333	
Pneumococcal vaccination	7,008	8,857	58,597	3,903	4,066	9,950	
Neuraminidase inhibitors	5,093	6,415	32,212	2,020	2,104	5,043	

^a^See Table 1.

Low and high levels for age-specific influenza vaccine efficacy show that the number of expected hospitalizations varies from almost 2,000 to >6,900 and the number of deaths varies from almost 800 to >2,800 (Table 7). These numbers are equal to a range of 30% to 80% in the percentage of the number of hospitalizations and deaths that might be avoided (base case 55% to 60%).

**Table 7:** Hospitalizations and deaths for alternative influenza vaccine efficacy^a^

Scenario	No. of hospitalizations			No. of deaths			
	Base case	Vaccine efficacy		Base case	Vaccine efficacy		
		All age groups equal to 80%^b^	Age groups <64 y = 40%; for >65 = 30%^c^		All age groups equal to 80%	Age groups <64 y = 40%; for >65 y = 30%^c^	
Nonintervention	10,186	10,186	10,186	4,040	4,040	4,040	
Influenza vaccination							
Total population	3,847	2,037	6,866	1,738	808	2,811	
Risk groups	3,968	2,158	6,926	1,789	859	2,837	

^a^See Table 1. ^b^Minimum variant based on Meltzer et al. (9). ^c^Maximum variant assumes 80% efficacy for all ages.

For the pneumococcal vaccine scenario, we tested two parameters: the percentage of complications (25% to 75%) to be prevented by pneumococcal vaccination and the pneumococcal vaccine efficacy (also 25% to 75%). Our results showed that the number of expected hospitalizations varies from 5,400 to 8,950, the number of deaths varies from >3,800 to 4,000 (Table 8). These values are equal to a range of 12% to 47% (base case 31%) and 1% to 5% (base case 3%) in the percentage of the number of hospitalizations and deaths that might be avoided. When assuming 25% to 75% effectiveness for the neuraminidase inhibitors scenario, we also estimated that between 25% and 75% of the number of hospitalizations and deaths can be avoided.

**Table 8:** Hospitalizations and deaths for alternative values for pneumococcal related variables^a^

Scenario	No. of hospitalizations					No. of deaths				
	Base case	Reduction of complications		Vaccine efficacy		Base case	Reduction of complications		Vaccine efficacy	
		25%	75%	25%	75%		25%	75%	25%	75%
Non intervention	10,186	10,186	10,186	10,186	10,186	4,040	4,040	4,040	4,040	4,040
Pneumococcal vaccination	7,008	8,597	5,418	8,945	7,703	3,903	3,971	3,834	3,986	3,933

^a^See Table 1.

## Discussion

The nonintervention scenario describes a pandemic situation in which no interventions are available; such an influenza pandemic, with a gross attack rate of 30%, would result in five times as many influenza-related hospitalizations and deaths as in a regular influenza epidemic with the current degree of vaccination, mostly in persons >65 years of age. Sensitivity analysis shows that varying the gross attack rate does not change the percentage of hospitalizations and deaths that might be avoided in the different scenarios. Varying the age-specific attack, hospitalization, and death rates has a large impact on the estimated number of hospitalizations and deaths. However, the impact is less in terms of the percentage of the number of hospitalizations and deaths that might be avoided by the various interventions.

Influenza vaccination may prevent many hospitalizations and deaths. The influenza vaccination scenario suggests that when assuming the age-specific complication rates of a regular epidemic, vaccination of the total population compared to vaccination of healthcare workers and the groups at risk for influenza would do little to avert hospitalizations and deaths. However, sensitivity analysis shows this result to be quite sensitive to the assumptions of the complication rates by age. As a consequence of higher complication rates in lower age and risk groups, the percentage of averted hospitalizations and deaths substantially decreases in the scenario’s pneumococcal and influenza vaccination of risk groups for influenza.

Only a pandemic itself can provide better estimates of the age-specific attack and complication rates, but these analyses show a range of what might be expected. While the likelihood of an available influenza vaccine in the beginning of a pandemic is low, the next best option seems to be the therapeutic use of neuraminidase inhibitors. However, this option has three major considerations: 1) effective use of neuraminidase inhibitors depends greatly on the assumption of 50% effectiveness to prevent hospitalizations and deaths; 2) every patient with influenzalike illness must begin medication within 48 hours after onset of symptoms (a logistically complicated task); and 3) a sufficient stock of neuraminidase inhibitors must be available, which is currently not the case. In our current approach, we probably underestimated the effect of influenza vaccination and the therapeutic use of neuraminidase inhibitors because we did not take into account the specific features of influenza as an infectious transmissible disease.

Pneumococcal vaccination could prevent 31% of the hospitalizations and 3.4% of the deaths. This intervention is the least effective because pneumococcal vaccination prevents only one complication of influenza (i.e., invasive pneumococcal infections). In contrast to hospitalizations, few deaths might be prevented by pneumococcal vaccination because relatively more excess hospitalizations than deaths are attributable to influenza-related pneumonia. An advantage of this intervention is that pneumococcal vaccination can be done before the pandemic starts since the vaccine is effective in preventing invasive pneumococcal infections for approximately 5 years (*15*). As expected, sensitivity analysis showed that lower vaccine effectiveness results in less hospitalizations and deaths prevented. In the next pandemic, if pneumoccocal infections occur more often as a complication of influenza than in the base case, using this intervention would prevent increased hospitalizations and deaths.

The objective of our study was to examine the potential impact (in terms of hospitalizations and deaths) of pandemic influenza in the Netherlands and to analyze the effects of several possible interventions. Ideally, after a pandemic has started, the influenza vaccine should be available and administered as quickly as possible following a prioritized scheme. In the Netherlands, developing this scheme is a governmental task. The scheme may be dependent on the actual (observed) age-specific attack and complication rates. However, at the start of the pandemic, no vaccine is expected to be available. Based on our analysis and assumptions, we conclude that a combined strategy of pneumococcal vaccination of risk groups for influenza together with the therapeutic use of neuraminidase inhibitors for all patients with influenzalike illness (within 48 hours after onset of symptoms) is the best strategy in preventing hospitalizations and deaths.

This recommendation is not valid if therapeutic use of neuraminidase inhibitors is shown to be ineffective in preventing influenza-related hospitalizations and deaths. Also, if the next pandemic shows that invasive pneumococcal infections are not a complication of influenza, pneumococcal vaccination is no longer a valid intervention. Because these questions are still unanswered, we also recommend ongoing research in the field of vaccine production techniques.

To prepare effectively for the next pandemic, the Dutch government will continue to investigate stockpiling neuraminidase inhibitors and securing influenza vaccine supply during a pandemic.

Our scenario analysis provides information about reducing the effects of a pandemic to a minimum, both regionally and nationally, to those who must prepare for the control of an actual pandemic. The insights from the scenario analysis provide a possible order of magnitude for providing healthcare (regional data were also calculated; data not shown). Furthermore, by using a model and a set of assumptions, we compared the effects of various interventions on the demand for care. Scenario analysis provided insight into which parameters have the most influence on the outcome variables (the age-specific attack and complication rates). If outbreaks of a new, potentially pandemic, influenza virus occur abroad and if these outbreaks yield real information about the attack and complication rates by age group, we can use these values in our model to update the estimate of the demand for care that can be expected in the Netherlands, nationally and regionally. Other countries might also use a similar approach to support their pandemic preparedness planning.
